# Transportation of whey protein-derived peptides using Caco-2 cell model and identification of novel cholesterol-lowering peptides

**DOI:** 10.29219/fnr.v67.9079

**Published:** 2023-05-25

**Authors:** Feifan Liu, Mingzhen Liu, Tao Zhang, Xuan Zhao, Xiaozhi Wang, Weimei Kong, Li Cui, Haibo Luo, Lili Guo, Yuxing Guo

**Affiliations:** 1 Department of Food Science and Technology, School of Food Science and Pharmaceutical Engineering, Nanjing Normal University, Nanjing, Jiangsu, PR China; 2 Institute of Agro-Product Processing, Jiangsu Academy of Agricultural Sciences, Nanjing, PR China; 3 College of Traditional Chinese Medicine and Food Engineering, Shanxi University of Chinese Medicine, Jinzhong, PR China

**Keywords:** whey protein, enzymatic hydrolysis, cholesterol-lowering peptides, Caco-2 cells, intestinal transport, gastrointestinal digestion

## Abstract

**Background:**

The increasing morbidity and mortality of cardiovascular disease have become a major factor in human death. Serum cholesterol is considered to be an important risk factor for inducing coronary heart disease, atherosclerosis and other cardiovascular diseases. To screen intestinal absorbable functional small peptides with cholesterol-lowering activity by enzymatic hydrolysis of whey protein and develop cholesterol-based functional food that may become a substitute for chemically synthesized drugs, providing new ideas for diseases caused by high cholesterol.

**Objective:**

This study aimed to evaluate the cholesterol-lowering activity of intestinal absorbable whey protein-derived peptides hydrolyzed by alkaline protease, trypsin and chymotrypsin, respectively.

**Method:**

The whey protein hydrolysates acquired by enzymatic hydrolysis under optimal conditions were purified by a hollow fiber ultrafiltration membrane with a molecular weight cutoff of 10 kDa. The fractions obtained by Sephadex G-10 gel filtration chromatography were transported through a Caco-2 cell monolayer. The transported peptides were detected in the basolateral aspect of Caco-2 cell monolayers using ultra- performance liquid chromatography-tandem mass spectrometry (UPLC-MS).

**Results:**

His-Thr-Ser-Gly-Tyr (HTSGY), Ala-Val-Phe-Lys (AVFK) and Ala-Leu-Pro-Met (ALPM) were unreported peptides with cholesterol-lowering activity. The cholesterol-lowering activities of the three peptides did not change significantly during simulated gastrointestinal digestion.

**Conclusion:**

This study not only provides theoretical support for the development of bioactive peptides that can be directly absorbed by the human body, but also provides new treatment ideas for hypercholesterolemia.

## Popular scientific summary

- This study may provide a theoretical basis for the development of the cholesterol-lowering functional food market.
- HTSGY, AVFK and ALPM could be completely transported across Caco-2 cell monolayers.
- HTSGY, AVFK and ALPM are unreported peptide sequences with cholesterol-lowering activity.
- Peptides HTSGY, AVFK, ALPM could be potential hypocholesterolemic agents with resistance to digestive enzymes.

Atherosclerosis, hypertension, and especially hypercholesterolemia caused by excessive cholesterol levels have significant negative effects on human health (1, 2). In recent years, high cholesterol disease has been treated through a combination of medication and dietary restrictions (3, 4). However, studies have demonstrated that the most commonly used cholesterol-lowering medicine causes adverse side effects (5–7). Nonetheless, many types of foods contain bioactive peptides that decrease serum cholesterol levels without side effects (8). Therefore, bioactive peptides have received increasing attention in reducing serum cholesterol levels. For instance, novel bovine casein-derived peptides could reduce cholesterol content by inhibiting the solubility of micellar cholesterol (9). Leu-Pro-Tyr-Pro-Arg (LPYPR) derived from soy peptides can be regarded as hypolipidemic functional peptide (10).

Currently, there are many studies on the effects of food-derived cholesterol-lowering peptides derived from soy and casein on serum cholesterol levels in animals or humans, while few studies on whey protein-derived cholesterol-lowering peptides. Nevertheless, whey protein-derived bioactive peptides have the potential of great research value (11–13). Baba et al. reported that new hypocholesterolemic bioactive peptides could be obtained by hydrolysis of camel whey protein (14). Morikawa et al. explored that lactostatin (a polypeptide prepared from β-lactoglobulin) has cholesterol-lowering activity while increasing CYP7A1levels and mRNA expression in mouse hepatocytes (15).

Nevertheless, one of the biggest challenges in developing whey protein peptides as functional food ingredients is to demonstrate the *in vivo* efficacy of the bioactive components. The potential utility of whey protein peptides depends on the ability to reach target organs after oral administration. Since peptide absorption occurs mainly in the small intestinal epithelium, a key factor affecting the bioactivity of bioactive peptides is their transport through the intestinal epithelium. Human colon adenocarcinoma cells line Caco-2 that exhibit the morphological and functional properties of mature enterocytes after differentiation has been utilized to predict the intestinal absorption of active ingredients (16–19). Ding et al. established a Caco-2 cell monolayer model to analyze the transport of ACE inhibitory peptide Thr-Asn-Gly-Ile-Ile-Arg (TNGIIR) in the small intestine and the mechanism of intestinal absorption (20). The GI digestive enzymes are another influencing factor for the function of bioactive peptides in the human body (21). To further explore whey protein cholesterol-lowering peptides resistant to gastrointestinal digestion, it is necessary to understand their stability in the gastrointestinal tract. Ruiz et al. demonstrated that the activity of most peptides found in Manchego cheese did not change drastically, except for the peptide Thr-Gln-Pro-Lys-Thr-Asn-Ala-Ile-Pro-Tyr (TQPKTNAIPY) from *α*s-2 casein, which exhibited a significant increase in activity after simulated digestion (22). Obviously, bioactive peptides in natural foods have different roles during gastrointestinal digestion. Accordingly, it would be more meaningful to establish a Caco-2 cell monolayer model and simulate the changes of bioactive peptide fractions in the gastrointestinal tract.

The purpose of the present project was to obtain intestinal absorbable whey protein-derived peptides with cholesterol-lowering activity. Firstly, whey protein hydrolysates were purified by a hollow fiber ultrafiltration membrane and Sephadex G-10 gel filtration chromatography. Secondly, the purified samples were transported through Caco-2 cells to obtain absorbed peptides, which were identified with UPLC-MS. Furthermore, the peptide sequences that may have cholesterol-lowering activity are synthesized and investigated for *in vitro* cholesterol- lowering activity. Finally, a two-stage *in vitro* digestion model system was used to simulate the human gastrointestinal digestion process.

## Materials and methods

### Materials

Whey protein concentrate powder was purchased from Hilmar Cheese Company (WPC-80, 90%; Suzhou, China). Alkaline protease, pepsin, trypsin, and chymotrypsin were acquired from Nanjing Odofoni Biotechnology Co., Ltd. (Nanjing, China). The Sephadex G-10 gel column was purchased from Amersham Pharmacia Biotech (1.0 × 40 cm, Uppsala, Sweden). Acetonitrile of chromatographic grade was obtained from Honeywell International (Morristown, NJ, USA). Trifluoroacetic acid (TFA) was purchased from Sigma-Aldrich (St. Louis, MO, USA). The Caco-2 human colorectal adenocarcinoma cell line was bought from the Institute of Biochemistry and Cell Biology (CAS; Shanghai, China). Transwell Permeable Supports, Cell culture medium, Dulbecco’s modified Eagle’s medium (DMEM), and trypsin were purchased from Hyclone Laboratories (Logan, UT). Hanks’ balanced salt solution (HBSS) was obtained from Gibco Invitrogen (Burlington, Canada). Alkaline phosphatase (AKP) assay kit (Nanjing Jiancheng Bioengineering Institute, Nanjing, China). The peptides His-Thr-Ser-Gly-Tyr (HTSGY), Ala-Val-Phe-Lys (AVFK) and Ala-Leu-Pro-Met (ALPM) with a purity of 90% were synthesized by Shanghai Peptide Co. (Shanghai, China).

### Production of whey protein hydrolysates

Whey protein powder was completely dissolved in deionized water to obtain a 6% (W/V) whey protein solution and heated at a constant temperature of 65°C for 15 min. After complete dissolution, the pH was adjusted to 8.5, 8.0 and 7.5, respectively. When the temperature dropped to the reaction temperatures, alkaline protease, trypsin, and chymotrypsin were added to the whey protein solution and incubated, respectively. The pH was adjusted by continuously adding 0.1 M NaOH solution to maintain the above optimal pH value. Furthermore, the consumption of NaOH concentration is recorded, which can be used to calculate the degree of hydrolysis (DH) according to the literature (23). After the hydrolysis, the whey protein hydrolysates were boiled for 20 min to denature the enzymes. The whey protein hydrolysate supernatant was collected by centrifugation (7000 *×g*, 10 min) and analyzed for peptide concentration according to the biuret reaction assay (24).

### Purification and identification of whey protein peptides

Whey protein hydrolysates were purified using hollow fiber ultrafiltration membranes to cut off peptides liquid under 10 kDa. The initial ultrafiltration membrane pressure was 0.15 Mpa with the flow rate of 5–10 mL/min. The fractions below 10 kDa were purified using a Sephadex G-10 column (25). The whey protein hydrolysate was applied to a Sephadex G-10 column equilibrated with distilled water. Peptides were eluted with distilled water at a flow rate of 3.5 mL/min. Elution curves were obtained by measuring absorbance at 220 nm. Fractions were collected at 2 min intervals. Fractions were combined and concentrated by lyophilization, which was stored at -20°C for further analysis.

### Establishment of Caco-2 cell monolayer model

Caco-2 cells were cultured in a high-glucose DMEM medium containing 10% fetal bovine serum and 1% penicillin-streptomycin mixture at 37°C, 5% CO_2_. Caco-2 cells were seeded in Transwell Permeable Supports (1.12 cm^2^, 0.4 μm pore size, Corning, NY, USA) at 2 × 10^5^ cells/cm^2^. A 0.5 mL of Caco-2 suspension was added in the Apical Side (AP) compartment of 12-well polycarbonate Transwell plates and 1.5 mL DMEM complete medium were seeded in the basal lateral (BL) compartment. After seeding, cells were cultured under the same conditions with medium changes every other day for 1 week and then daily thereafter until a monolayer formed.

The transepithelial resistance (TEER) value was determined using a Millicell ERS-2 Voltohmmeter (Millipore, Bedford, MA). Cell permeability was measured in three parallels. On days 3, 10, 15 and 21 days, AKP, which is the hallmark membrane-bound glycoprotein of the brush-border cells of the intestinal epithelium when cells begin to differentiate, was measured using a commercial AKP assay kit (5). The volume was 0.5 mL sodium fluorescein (0.5 g/L) on the AP side, 1.5 mL HANKS (pH 7.4) was added on the BL side, and the absorbance on the BL side was measured at excitation 485 nm – emission 535 nm to evaluate the integrity of the Caco-2 monolayer (26).

### Transportation and identification of whey peptides in the Caco-2 cell monolayers

The Caco-2 cell monolayers model cultured in Transwell plate was washed twice with HANKS (pH 7.4) after formation, which was incubated with HANKS buffer (pH 7.4) for 30 min in a 37°C, 5% CO_2_ incubator. The whey protein-derived peptides fraction from the Sephadex G-10 column separation was adjusted to 10 mg/mL using HANKS buffer (pH 7.4) and added to the AP chamber, and 1.5 mL of HANKS buffer (pH 7.4) was added to the BL side. After 1 h of incubation, the solution was removed from the BL wells and filtered through a 0.22 μm water filter. The BL solution was collected and analyzed using UPLC-MS. The molecular mass of peptides was determined using a Waters MALDI-Q-TOF MS (matrix-assisted laser desorption/ ionization quadrupole-time-of-flight mass spectrometer, Waters Corporation, Manchester, UK). The MS/MS amino acid sequence was carried out by GPMAW software and Masslynx software.

### Bioinformatics prediction of synthetic peptides

The resistance of transit peptides to digestion was analyzed using the program ExPASy peptide cutter (https://web.expasy.org/peptidecutter/). Prediction of peptide hydrophobicity with ProtScale (https://web.expasy.org/cgibin/protscale/protscale.pl#opennewwindow). All peptides were screened for bioactive peptides through the MBPDB database (http://mbpdb.nws.oregonstate.edu/).

### Peptide synthesis and cholesterol-lowering activity assay

The cholesterol-lowering activity was measured using synthesized peptides (AVFK, ALPM, and HTSGY) of approximately 90% purity. The solubility of micellar cholesterol was measured after the addition of 1 mg/mL of each peptide fraction to an *in vitro* prepared intestinal micelle suspension with reference to the method of Jiang et al. with some modifications (9). Sodium phosphate buffer at pH 7.4 containing 2 mmol/L cholesterol and 5 mmol/L linoleic acids was mixed with 135 mmol/L NaCl and 10 mmol/L sodium taurocholate. The suspension was ultrasonicated twice for 2 min at 95% energy output (100 W) using a Misonix 3000 ultrasonicator (Misonix, New York, NY) to form homogeneous micelles. The supernatant fraction was filtered through a 0.22-μm Millex-GP filter (Millipore, Bedford, MA). The supernatant fractions (50 µL) were collected, and the cholesterolconcentrations of supernatant was measured the absorbance at 510 nm according to Total Cholesterol (T-CHO)Assay Kit (Nanjing Jiancheng Institute of BiologicalEngineering, Nanjing, China). Each experiment was performed in triplicate and cholesterol concentrations and cholesterol-lowering effect were calculated according to formulae [1] and [2], respectively.


$$Concentration\;of\;cholesterol\;\left(\frac{mmol}{l}\right)=\frac{OD_{2}-OD_{0}}{OD_{1}-OD_{0}}\times concentration\;of\;calibrator\;\left(\frac{mmol}{l}\right)$$ [1]

where OD_0_ is the absorbance of the blank measured by optical density, OD_1_ is the absorbance of the standard and OD_2_ is the absorbance of the sample:


$$Inhibition\;of\;micellar\;cholesterol\;solubility\left(\%\right)=\frac{C_{0}–C_{2}}{C_{0}}\times 100\%$$ [2]

where C_0_ is the cholesterol concentration of the original micelles and C_2_ is the cholesterol concentration after the addition of the peptide fraction.

### Simulation of GI digestion

*In vitro* digestion simulations were performed using a slight modification of the two-stage hydrolysis method published by Ruiz (22). Hydrolysates were prepared from an aqueous solution of the synthetic peptides (AVFK, ALPM, and HTSGY) (10 mg/mL). The peptides were first hydrolyzed with NaCl and pepsin (1:60000, 3400 units/mg) (enzyme: substrate ratio of 1:50 w/w) at 37°C, pH 2.0 for 90 min, and then the pH was adjusted to 7.0 using NaOH 1 M and the sample was hydrolyzed with Corolase PP^®^ (enzyme: substrate ratio of 1:25 w/w) at 37°C. Corolase PP^®^ is a proteolytic enzyme preparation from the porcine pancreas that contains a variety of amino and carboxy peptidase activities in addition to trypsin and chymotrypsin. Hydrolysis was carried out in a constant temperature water bath with constant stirring. The reaction was quenched by heating in a water bath at 95°C for 10 min. Each sample was stored at -20°C until further analysis.

### High-performance liquid chromatography analysis and bioavailability determination

High-performance liquid chromatography (HPLC) analysis of undigested samples and digests was performed using an Agilent 1100 high-performance liquid chromatograph equipped with an HPLC column (Agilent ZOREAX 300 SB-C18, 4.6 × 250 mm, 5 μm, Agilent Technologies, USA). The mobile phase consisted of 0.05% TFA aqueous solution (A) and 0.05% TFA acetonitrile solution (B). The detection wavelength was 214 nm and the column temperature was set at 30°C. The elution of the column was completed at a constant flow rate of 1 mL/min with the following gradient from 0 to 5 min using 5% B, increasing to 25% B at 15 min, and then remaining at 25% B until 20 min.

The bioavailability of the peptides was determined by peak area contents according to the HPLC profiles. The peptide content of the fractions and their digests were manifested by the peak areas.


$$Bioavailability\;\left(\%\right)=\frac{\begin{array}{l} peak\;area\;of\;the\;peptide\;\\ remaining\;after\;GI\;digestion \end{array}}{initial\;peptide\;peak\;area\;}\times \;100\%$$ [3]

### Effects of AVFK, ALPM, and HTSGY on cholesterol absorption in Caco-2 cells in vitro

Caco-2 cells were digested, centrifuged and counted at a final concentration of 5.0 × 10^4^ cells/well. A 100 µL of the cell suspension was inoculated into 96-well plates and incubated at 37*°* in a 5% CO_2_ incubator for 24 h. The original culture medium was discarded and five different concentrations (0, 0.5, 1.0, 1.5, and 2 mg/mL)of synthetic peptides (AVFK, ALPM, and HTSGY) were added. The cell viability was determined according to the 3-(4,5-dimethylthiazol-2-yl)-2,5-diphenyltetrazolium bromide (MTT) assay of Jiang et al. (9).

Different concentrations (0, 0.5, 1.0, 1.5 and 2 mg/mL) of AVFK, ALPM and HTSGY were dissolved in sterile PBS and then sterilized using a 0.22 µm microporous filter. The cultured cells were washed three times with PBS solution. A 900 μL of cholesterol solution and 100 μL of different concentrations of AVFK, ALPM and HTSGY solutions were added to the AP side and 1.5 mL of DMEM culture solution to the BL side; 1 mL of cholesterol solution was added to the AP side of the blank control group. After 24 h of incubation at 37°C and 5% CO_2_, the solution in the wells was discarded and washed three times with 1 mL of PBS solution. Caco-2 cells were digested with trypsin and the cell pellet was collected by centrifugation at 1,000 × g for 10 min at 4°C. Caco-2 cell pellets were washed twice with 0.1 mol/L phosphate buffer (pH 7.2) and centrifuged at 1,000 × g for 10 min at 4°C. Cells were resuspended in 0.3 mL of 0.1 mol/L phosphate buffer (pH 7.2) and sonicated in an ice- water bath (300 W, 5 s each at 30 s intervals, repeated five times). Absorbance was measured at 510 nm according to the total cholesterol kit (Nanjing Jiancheng Institute of Biological Engineering, Nanjing, China) and the cholesterol content of the cells was calculated according to equation (1).

### Statistical tests

The data analysis was performed with Prism 6.0 (GraphPad) and one-way ANOVA (SPSS Statistics 20) followed by Dunnett’s test. Statistical results were presented as mean ± standard deviation (X ± SD, *n* = 3). Differences were considered significant at *P* < 0.05.

## Results and discussion

### Preparation and purification of whey protein peptides

**Fig. 1:**
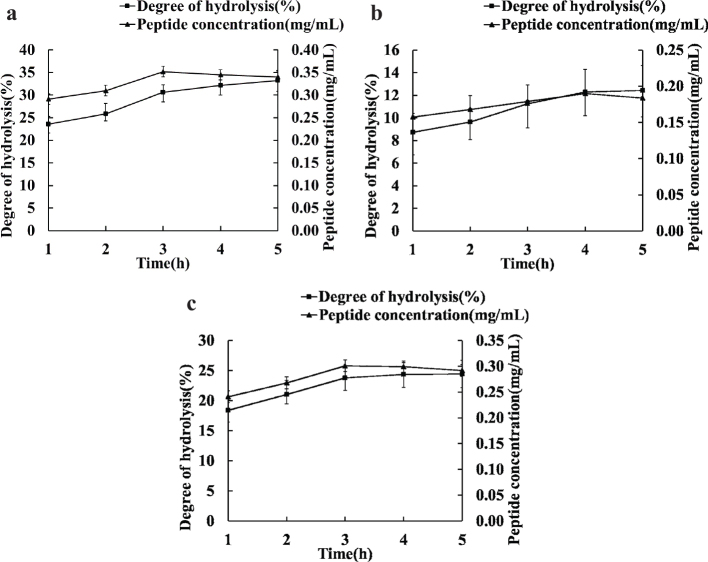
Plot of the degree of whey protein hydrolysis and peptide concentration. (a–c) represent the effects of alkaline protease, trypsin and chymotrypsin on whey protein hydrolysis degree and peptide concentration.

The optimal hydrolysis conditions were evaluated by measuring peptide content and the DH value during hydrolysis of whey protein by three proteases. As shown in Fig. 1, peptide concentration and DH value increased in the first 3 h and then tended to be stable with the increase of enzymatic hydrolysis. The peptide concentration and DH value of whey protein-derived peptides hydrolysate by alkaline protease were much higher than those of peptides hydrolyzed by trypsin and chymotrypsin. Probably because trypsin is the specific protease, which can only selectively hydrolyze peptide bonds composed of carboxyl groups of Lys (K) or Arg (R) in proteins. Chymotrypsin is a stronger hydrolyzer than trypsin, which acts mainly on the carboxyl groups of Tyr (Y), Trp (W) and Phe (F) (27). However, the main component of alkaline protease is a serine protease that cleaves a wide range of hydrophobic amino acids terminating in Ile (I), Leu (L) and Val (V) (28). Our results showed that the cleavage site of the protease affects peptide concentration and the DH value of hydrolysate.

**Fig. 2:**
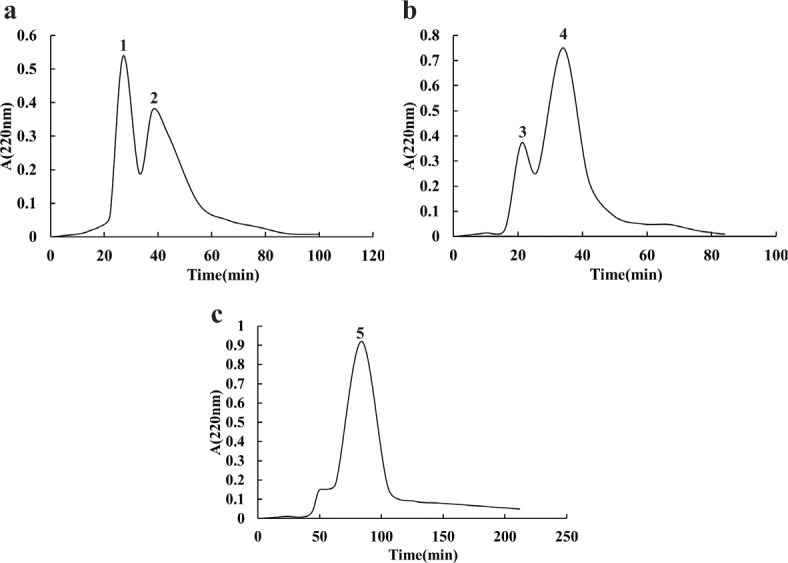
Sephadex G-10 column for purification of protein hydrolysates. (a) (fractions 1, 2), (b) (fractions 3, 4), and (c) (fractions 5) represent alkaline protease, trypsin and chymotrypsin hydrolysates purified by the Sephadex G-10 column.

Different whey protein-derived peptides hydrolyzed by alkaline protease, trypsin and chymotrypsin were selected for subsequent ultrafiltration membranes with a 10 kDa cutoff. To screen the small molecular weight peptides, the MW < 10 kDa fraction was separated using Sephadex G-10 gel filtration chromatography. Two peaks (samples 1 and 2) were produced from the alkaline protease hydrolysate (Fig. 2a). Samples 3 and 4 are trypsin hydrolysates (Fig. 2b), and only one subfraction (sample 5) was obtained by chymotrypsin hydrolysis (Fig. 2c). Five fractions (samples 1, 2, 3, 4, and 5) were collected and lyophilized separately for subsequent Caco-2 cell transport experiment.

### Establishment of an intestinal Caco-2 cell monolayer model

On the 21st day, the electron microscope section of the Caco-2 cell monolayer clearly showed that the microvilli structure on the upper side of the cell monolayer had been differentiated and the brush-like border was very neat and dense (Fig. S1A). The TEER value was 520 Ω·cm^2^ (Fig. S1B), and the AKP activity ratio (AP/BL) (Fig. S1C) was determined to be 0, 0.99, 1.64 and 2.11 on days 3, 10, 15 and 21. Caco-2 cell monolayers to be used for transport studies have TEER values greater than 500 Ω·cm^2^, indicating that the modeling was successful (29). In addition, the permeability of sodium fluorescein in the experimental group was significantly lower than that in the blank control group (Fig. S1D).

### Whey peptides absorbed by Caco-2 cell monolayer model

**Fig. 3:**
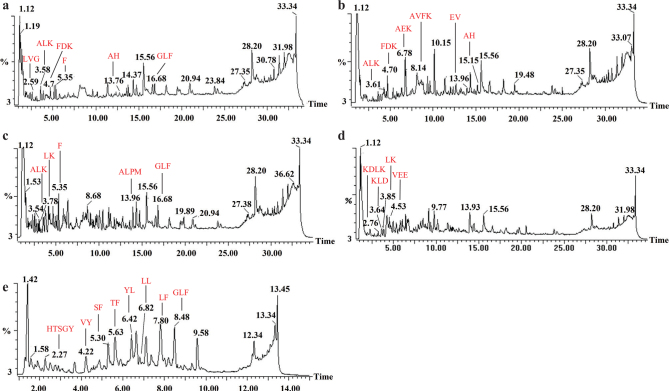
The TIC chromatograms of the different fractions after passing through the Caco-2 cell monolayer. (a–e) stand for TIC chromatograms of fractions 1, 2, 3, 4 and 5 after being transported through the Caco-2 cell monolayer, respectively.

**Fig. 4:**
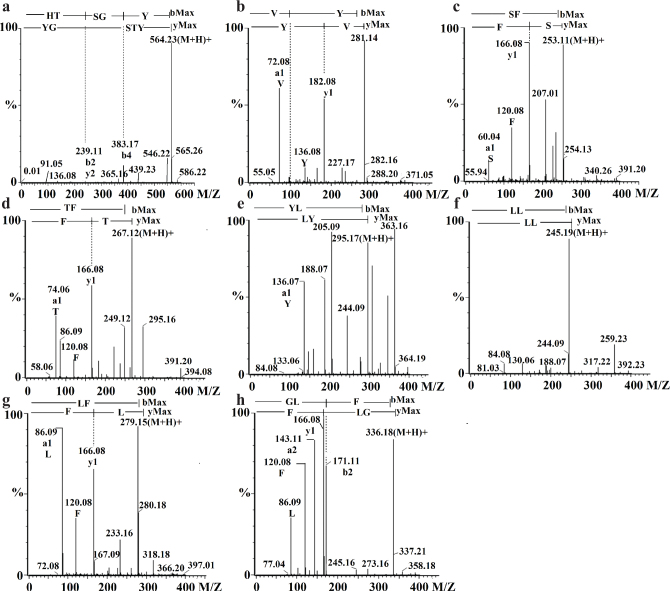
Tandem mass spectra of absorbable peptides in fraction 5. (a–h) represent HTSGY, VY, SF, TF, YL, LL, LF and GLF in the order.

Peptide sequences were identified by UPLC-MS after five samples (fractions 1, 2, 3, 4, and 5) were transported through Caco-2 cell monolayers, respectively. The total ion chromatograms (TICs) of fractions 1, 2, 3, 4, and 5 after across a Caco-2 cell monolayer are shown in Fig. 3a–e. From fraction 1, six whey protein-derived peptides at retention times (RT) 2.59, 3.58, 4.70, 5.35, 13.76, and 16.86 were identified as Leu-Val-Gly (LVG), Ala-Leu-Lys (ALK), Phe-Asp-Lys (FDK), Phe (F), Ala-His (AH) and Gly-Leu-Phe (GLF) (Table 1 and Fig. S2). From peak 2, most of the shorter peptides were eluted in the first 20 min and peaks were illustrated at RT of 3.61, 4.70, 6.78, 8.14, 13.96, and 15.15. These peptides were indicated as Ala-Leu-Lys (ALK), Phe-Asp-Lys (FDK), Ala-Glu-Lys (AEK), Ala-Val-Phe-Lys (AVFK), Glu-Val (EV), and Ala-His (AH) (Table 2 and Fig. S3). Based on the RT, peak 3 was identified with five peptides as ALK, Leu-Lys (LK), F, Ala-Leu-Pro-Met (ALPM), and GLF (Table 3 and Fig. S4). The RT of 2.76, 3.64, 3.85, and 4.53 were identified as Lys-Asp-Leu-Lys (KDLK), Lys-Leu-Asp (KLD), LK and Val-Glu-Glu (VEE) (Table 4 and Fig. S5). Peaks 2.27, 4.22, 5.30, 5.63, 6.42, 6.82, 7.80, 8.48 were showed from Fraction 5. These peptides were sequentially identified as His-Thr-Ser-Gly-Tyr (HTSGY), Val-Tyr (VY), Ser-Phe (SF), Thr-Phe (TF), Tyr-Leu (YL), Leu-Leu (LL), Leu-Phe (LF), and GLF using MALDI Q-TOF MS (Table 5, Fig. 4). The abundance of other unidentified peptides may have been too low to be analyzed in Fig. 3a–e by MALDI Q-TOF MS.

**Table 1:** Characteristics of peptides hydrolyzed from whey protein of fraction 1

RT (min)	Peptide sequence	Mr (Da)	[M+ H]^+^ m/z	GRAVY	Electrostatic Charge (pH = 7.0)	Source
2.59	LVG	287.36	288.2	2.53	0	*α*-lactalbumin (9–11)
3.58	ALK	330.42	331.3	0.57	1.0	*β*-lactoglobulin (155–157)
4.7	FDK	408.45	409.3	-1.53	0	*β*-lactoglobulin (152–154)
5.35	F	165.2	166.1	2.8	0	*α*-lactalbumin (4,14,28,50,72,99) *β*-lactoglobulin (98,152,167)
13.76	AH	226.23	227.2	-0.7	0.1	*α*-lactalbumin (125–126)
16.86	GLF	335.4	336.2	2.07	0	*α*-lactalbumin (70–72)

**Table 2:** Characteristics of peptides hydrolyzed from whey protein of fraction 2

RT (min)	Peptide sequence	Mr (Da)	[M+ H]^+^ m/z	GRAVY	Electrostatic charge (pH = 7.0)	Source
3.61	ALK	330.42	331.3	0.57	1.0	β-lactoglobulin (155–157)
4.7	FDK	408.45	409.3	-1.53	0	β-lactoglobulin (152–154)
6.78	AEK	346.38	347.2	-1.87	0	β-lactoglobulin (89–91)
8.14	AVFK	463.57	464.3	1.23	1	β-lactoglobulin (96–99)
13.96	EV	246.26	247.1	0.35	-1	α-lactalbumin (26–27)
15.15	AH	226.23	227.2	-0.7	0.1	β-lactoglobulin (143–144)

**Table 3:** Characteristics of peptides hydrolyzed from whey protein of fraction 3

RT (min)	Peptide sequence	Mr (Da)	[M+ H]^+^ m/z	GRAVY	Electrostatic charge (pH = 7.0)	Source
3.54	ALK	330.42	331.3	0.57	1.0	*β*-lactoglobulin (155–157)
3.78	LK	259.35	260.2	-0.05	1.0	*α*-lactalbumin (31–32)
5.35	F	165.2	166.1	2.8	0	*α*-lactalbumin (4,14,28,50,72,99) *β*-lactoglobulin (98,152,167)
13.96	ALPM	430.56	431.3	1.475	0	*α*-lactalbumin (34–35)
16.86	GLF	335.4	336.2	2.07	0	*β*-lactoglobulin (62–63)

**Table 4:** Characteristics of peptides hydrolyzed from whey protein of fraction 4

RT (min)	Peptide sequence	Mr (Da)	[M+ H]^+^ m/z	GRAVY	Electrostatic Charge (pH = 7.0)	Source
2.76	KDLK	502.61	503.4	-1.875	1.0	*α*-lactalbumin (32–35)
3.64	KLD	374.43	375.3	-1.2	0	*α*-lactalbumin (133–135)
3.85	LK	259.35	260.2	-0.05	1.0	*α*-lactalbumin (31–32) *α*-lactalbumin (34–35) *β*-lactoglobulin (62–63) *β*-lactoglobulin (156–157)
4.53	VEE	375.37	376.2	-0.93	-2	*β*-lactoglobulin (59–61)

**Table 5:** Characteristics of peptides hydrolyzed from whey protein of fraction 5

RT (min)	Peptide sequence	Mr (Da)	[M+ H]^+^ m/z	GRAVY	Electrostatic Charge (pH = 7.0)	Source
2.27	HTSGY	502.61	564.2	-1.28	0.1	*α*-lactalbumin (51–55)
4.22	VY	374.43	281.1	1.46	0	*β*-lactoglobulin (57–58)
5.30	SF	252.27	253.1	1.0	0	*α*-lactalbumin (3–4) *β*-lactoglobulin (166–167)
5.63	TF	266.29	267.1	1.05	0	*α*-lactalbumin (49–50)
6.42	YL	294.35	295.2	1.25	0	*β*-lactoglobulin (118–119)
6.82	LL	244.33	245.2	3.8	0	*α*-lactalbumin (7–8) *β*-lactoglobulin (4–5) *β*-lactoglobulin (47–48) *β*-lactoglobulin (73–74) *β*-lactoglobulin (119–120)
7.80	LF	278.35	279.1	3.3	0	*α*-lactalbumin (13–14) *α*-lactalbumin (71–72)
8.48	GLF	335.4	336	2.07	0	*α*-lactalbumin (70–72)

Notes: RT represents retention time; Red represents intact absorbable peptides matching the enzyme cut site; Green represents peptide sequences partially matching the enzyme cut site; Black represents small peptides identified that do not match the protease restriction site; Mr represents molecular weight; Electrostatic charge calculated from http://www.innovagen.se/index.asp; GRAVY calculated from www.gravy-calculator.de: positive value represents.

The majority of whey protein-derived peptides were observed to transport through the Caco-2 cell monolayer was in the dipeptide and tripeptide form (Tables 1–5), except for AVFK, ALPM, KDLK and HTSGY. It has been found that peptides obtained from protein hydrolysis (2–7 amino acids) were considered to be absorbed peptides and some could be absorbed in their intact form (30). Peptides of low molecular weight are more likely to transport across the intestinal barrier and exert biological effects than those of high molecular weight peptides (31). Furthermore, the transportation of whey protein-derived peptides may also be determined by hydrophilicity or the charge of peptides (32). For example, ALPM and AVFK are hydrophobic peptides (Tables 2 and 3). Studies have highlighted that hydrophobic peptides were primarily adsorbed to the surface of AP cell membranes through hydrophobic interactions and through hydrophobic interactions, by which the peptides were more readily transported and absorbed by the intestinal (33). The HTSGY is a positively charged peptide (Table 4). A peptide with a net positive charge enhances the overall absorption in the intestine (34). Consequently, it is presumed that the peptides that could be transported are related to molecular weight, hydrophobicity and charge, primarily related to molecular weight.

### Comparison of the specificity of the cleavage of three proteases

**Fig. 5:**
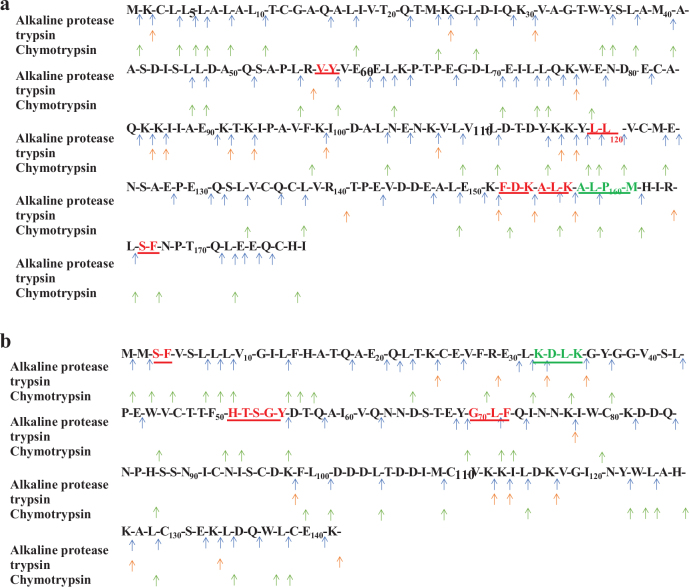
Map of the corresponding cleavage sites of different proteases in *β*-lactoglobulin and *α*-lactalbumin. (a, b) are the corresponding cleavage sites of alkaline protease, trypsin and chymotrypsin in *β*-lactoglobulin and *α*-lactalbumin, respectively; Identified peptides that can be transported by Caco-2 cells are marked with underline.

By analyzing the amino acid sequences of the peptides corresponding to the three protease cleavage sites, some peptides might be obtained by enzymatic digestion in the intact form by Caco-2 cells, while some peptides were produced by enzymatic digestion followed by peptidase hydrolysis of the brush border membrane (Fig. 5). Alkaline protease has a wide range of cleavage sites, trypsin is a very specific protease with cleavage sites for Lys (K) and Arg (R). Chymotrypsin is a very low specificity protease with cleavage sites for Phe (F), Tyr (Y), Trp (W), Leu (L), and Met (M). Hence, these three highly differentiated enzymes were selected to hydrolyze whey proteins for obtaining different forms of peptides.

We discovered that the restriction sites of peptides in fraction 1 and fraction 2 (ALK and FDK) were identical to the enzymatic cleavage site K of alkaline proteases by comparing the sequences of the amino acid and milk peptide libraries (Tables 1 and 2). The ALK in fraction 3 was derived from the fragment sequence KALKA of β-lactoglobulin, which matches one of the trypsin cleavage sites K (Table 3). After being transported to the Caco-2 cell model, SF, LL, GLF and HTSGY from *α*-lactalbumin were also identified, which matched the chymotrypsin enzymatic cleavage site (Table 5), indicating that they can be transported in the intact form. In addition, the amino acid sequences that partially matched three enzymatic cleavage sites appeared in fractions 3 and 4 (ALPM and KDLK) and the other peptides identified that did not match the protease restriction sites probably due to the action of the peptidase present on the brush border of Caco-2 cells to further hydrolyze the oligopeptides into amino acids, dipeptides or tripeptides (35) (Tables 3 and 4).

### Effect of ALPM, AVFK and HTSGY peptides on cholesterol-lowering activity in vitro

**Fig. 6:**
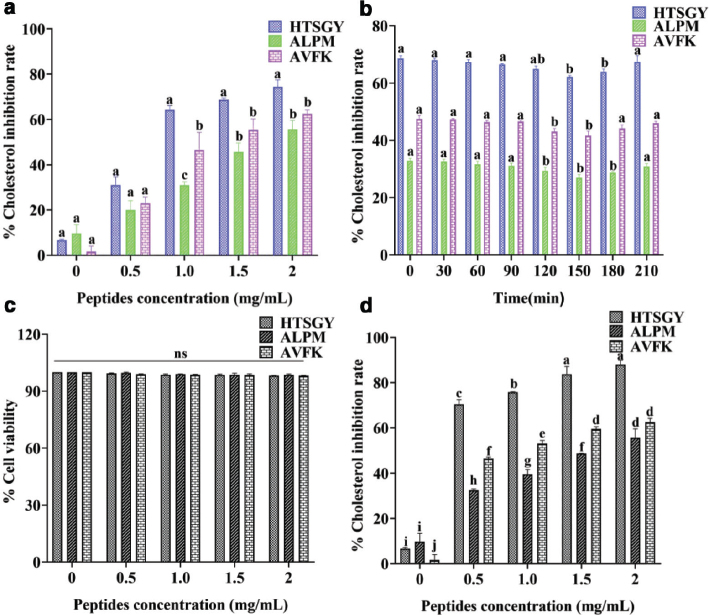
Effect of different concentrations of peptides on cholesterol absorption. (a) represents the determination of the cholesterol-lowering capacity of synthetic peptides HTSGY, ALPM, and AVFK. (b) represents the change in the cholesterol-lowering capacity of synthetic peptides HTSGY, ALPM, and AVFK during simulated gastrointestinal (GI) digestion. (c) represents Caco-2 cell viability after treatments with peptides HTSGY*,* ALPM and AVFK: results of MTT cell viability assay of Caco-2 cells after peptides treatments for 24 h. (d) represents Effects of different concentrations of peptides on water-soluble cholesterol uptake in Caco-2 cells. Notes: The data were expressed as mean ± standard deviation (SD, *n* = 3). Different lowercase letters in Figure A indicate significant differences within the same peptide concentration (*P* < 0.05); different lowercase letters in (b) indicate significant differences within the same peptide (*P* < 0.05) (Tukey test for one-way ANOVA, SPSS Statistics 20).

Cholesterol-lowering peptides were shown to have specific hydrophobic regions (36). Peptides containing hydrophobic amino acids such as Pro (P), Ala (A), or Tyr (Y) may have cholesterol-lowering abilities (37, 38). Since ALPM, AVFK, and HTSGY satisfy these characteristics, the synthetic peptides ALPM, AVFK and HTSGY were selected for their potential cholesterol-lowering ability by predicting the activity of the transport peptide in Caco-2 cells. As shown in Fig. 6a, the peptides ALPM, AVFK and HTSGY had inhibitory effects on the dissolution of cholesterol. The inhibition rate of AVFK and ALPM was (46.60 ± 5.45) % and (30.98 ± 1.03) % at a concentration of 1 mg/mL, respectively. The inhibition rate of HTSGY was significantly higher than that of the others, which was (64.27 ± 1.30) %.

The peptides ALPM and AVFK, which contain hydrophobic amino acids coalesce with non-polar molecules such as cholesterol, achieving a cholesterol-lowering effect by preventing cholesterol from dissolving into micelles. This mechanism is consistent with the cholesterol-lowering studies of peptides derived from rice bran protein (39). Conversely, the hydrophilic peptide HTSGY might reduce micellar cholesterol solubility by binding to micellar hydrophilic bile salts. It has been reported that hydrophilic peptides interact with the more polar micellar component bile salts to disrupt the formation of lipid micelles, thereby reducing cholesterol absorption (40).

### HPLC profiles and changes in bioavailability and cholesterol-lowering activity of ALPM, AVFK and HTSGY peptides during GI digestion

**Fig. 7:**
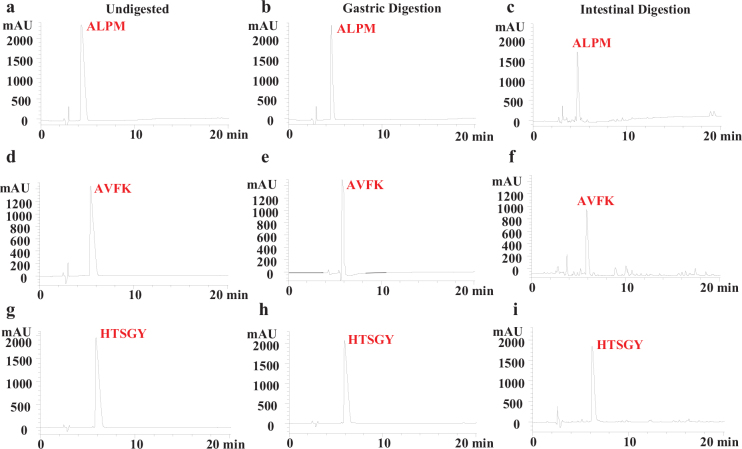
The HPLC profiles of synthetic peptides before and after simulated GI digestion. (a) undigested ALPM; (b) ALPM after gastric digestion; (c) ALPM after intestinal digestion; (d) undigested AVFK; (e) AVFK after gastric digestion; (f) AVFK after intestinal digestion; (g) undigested HTSGY; (h) HTSGY after gastric digestion; (i) HTSGY after intestinal digestion.

The cholesterol-lowering activity of three synthetic peptides ALPM, AVFK and HTSGY peptides were investigated during GI digestion (Fig. 6b). After digestion, the cholesterol-lowering activity of peptides ALPM, AVFK and HTSGY decreased by (5.43 ± 0.87) %, (13.32 ± 1.30) and (9.52 ± 0.89) %, respectively. The HPLC profiles of AVFK (Fig. 7a–c), ALPM (Fig. 7d–f) and HTSGY (Fig. 7g–i) during GI digestion showed that the hydrolysis of three peptides in simulated gastric digestion was not evident. After simulated intestinal digestion*,* the bioavailability of ALPM, AVFK and HTSGY was, respectively, 68.19, 72.16 and 83.66%. These results indicate that three synthetic peptides have a certain resistance to gastrointestinal enzymes and maintained cholesterol-lowering activity during GI digestion.

**Fig. 8:**
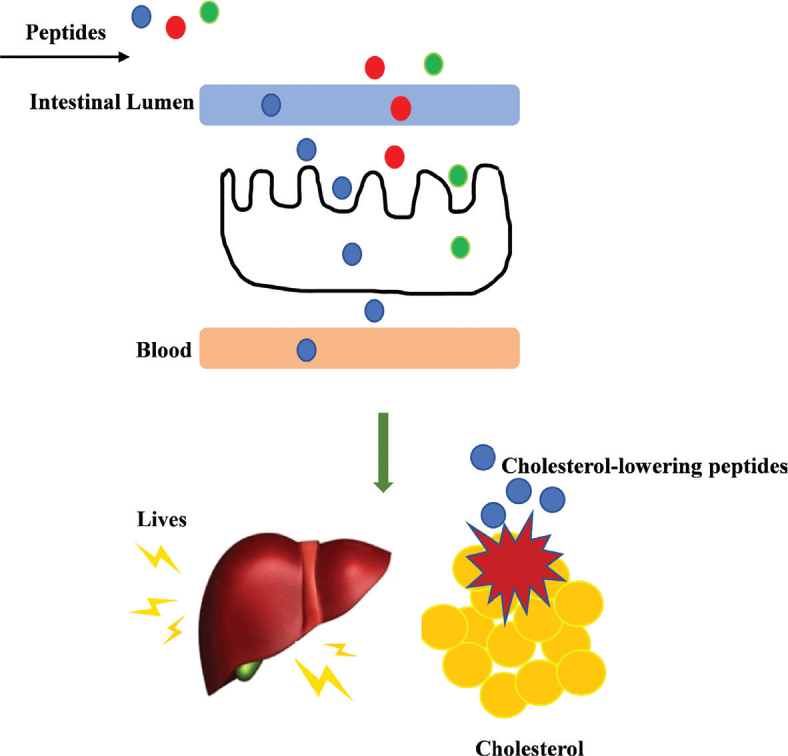
TOC chart.

Pepsin preferentially cleaves the C-terminus of Pro (P) and Leu (L) (Liang et al., 2018), and ALPM, AVFK and HTSGY were largely unhydrolyzed during simulated gastric digestion (Fig. 7b, e, h), probably because of the absence of preferential cleavage sites in the three peptides. Trypsin cleaved at the C-terminus of Lys (K) and Arg (R) residues, and chymotrypsin cleaved at the C-terminus of hydrophobic residues Phe (F), Tyr (Y), Trp (W) and Leu (L), resulting in the release of amino acids from ALPM, AVFK and HTSGY during simulated intestinal digestion (Fig. 7c, f, i). The elevated cholesterol-lowering activity during intestinal digestion might be associated with an increase in hydrophobic amino acids, whose increased levels may improve their ability to bind conjugated bile acids (41). It was noteworthy that peptides with Pro residues were resistant to degradation by digestive enzymes (42) and hydrophobic amino acids were slightly less resistant to gastrointestinal digestion (43). The HTSGY contains a large number of hydrophilic amino acids and ALPM contains Pro (P), hence HTSGY and ALPM might contribute better digestive resistance than AVFK.

### ALPM, AVFK and HTSGY peptides affect cholesterol absorption in Caco-2 cells

Through MTT experiments, we found that the cell survival rate was no less than 97% at peptide concentrations ranging from 0 to 2 mg/mL, which could exclude the potential toxic effects of the peptides on the Caco-2 cell line. Cell survival was higher than 99% at a concentration of 0.5 mg/mL for the three peptides, which did not affect cell growth (Fig. 6c). Therefore, in the following experiments aimed at studying the functional effects of peptides, the optimum concentration of the three peptides was 0.5 mg/mL.

In addition, we performed a preliminary study on the inhibition of water-soluble cholesterol by ALPM, AVFK and HTSGY in Caco-2 cell monolayers. After 2 h of the Caco-24 cell uptake experiment, the blank control had a cholesterol concentration in Caco-2 cells of 0.1213 mg/mL at 0.5, 1.0, 1.5 and 2.0 mg/mL, HTSGY, AVFK and AKPM significantly inhibited cholesterol uptake in Caco-2 cells. at 2.0 mg/mL, HTSGY was most effective in reducing cholesterol uptake was most effective at 2.0 mg/mL, with an inhibition rate of 88.72% (Fig. 6d). Interestingly, at 0.5 mg/mL HTSGY, the inhibition rate on Caco-2 cell monolayers absorption was over 50%, significantly higher than that of ALPM and AVFK at 32.15 and 46.52% (*P* < 0.05). The inhibition of monolayer cholesterol uptake by Caco-2 cells by HTSGY was significantly greater than that by 0.5 mg/mL at 1 mg/mL (*P* < 0.05). The inhibition by HTSGY and AVFK at 1.5 and 2.0 mg/mL was not statistically significant (*P* > 0.05). Overall, the inhibitory effect of peptide concentration on cholesterol uptake in Caco-2 cells had a dose-dependent effect.

## Conclusions

In this experiment, whey protein-derived peptides were hydrolyzed with alkaline protease, trypsin and chymotrypsin, respectively, and then transported through Caco-2 cell monolayers to screen intestinal absorption peptides. Among transported peptides, HTSGY, ALPM, and AVFK are found to be the unreported sequence that has cholesterol-lowering activity and resistance to gastrointestinal digestion. The study provided a research basis for the development of cholesterol-lowering peptide components for the functional food market.

## Authors’ contributions

Writing – original draft, Conceptualization, Methodology, Formal analysis, Investigation, Visualization and Data curation: F. F. L.; Validation, Resources, Writing – review and editing: M. Z. L, T. Z. and X. Z.; Writing – review and editing: X. Z. W., W. M. K., L. C. and H. B. L.; Validation, Resources, Writing – review and editing, Supervision: Y.X.G and L.L.G. All authors have read and agreed to the published version of the manuscript.

## Data Availability

Data are contained within the article.

## References

[1] Gaudet D, Drouin-Chartier JP, Couture P. Lipid metabolism and emerging targets for lipid-lowering therapy. Can J Cardiol 2017; 33(7): 872–82. doi: 10.1016/j.cjca.2016.12.019 28365054

[2] Liong MT, Shah NP. Acid and bile tolerance and cholesterol removal ability of lactobacilli strains. J Dairy Sci 2005; 88(1): 55–66. doi: 10.3168/jds.S0022-0302(05)72662-X 15591367

[3] Cannon CP. Low-density lipoprotein cholesterol: lower is totally better. J Am Coll Cardiol 2020; 75(17): 2119–21. doi: 10.1016/j.jacc.2020.03.033 32209335

[4] Edel AL, Aliani M, Pierce GN. Stability of bioactives in flaxseed and flaxseed-fortified foods. Food Res Int 2015; 77: 140–55. doi: 10.1016/j.foodres.2015.07.035

[5] Srinivasan B, Kolli AR, Esch MB, Abaci HE, Shuler ML, Hickman JJ. TEER measurement techniques for in vitro barrier model systems. J Lab Autom 2015; 20(2): 107–26. doi: 10.1177/2211068214561025 25586998 PMC4652793

[6] American Diabetes A. 3. Foundations of care and comprehensive medical evaluation. Diabetes Care 2016; 39 Suppl 1: S23–35. doi: 10.2337/dc16-S006 26696676

[7] Nijjar PS, Burke FM, Bloesch A, Rader DJ. Role of dietary supplements in lowering low-density lipoprotein cholesterol: a review. J Clin Lipidol 2010; 4(4): 248–58. doi: 10.1016/j.jacl.2010.07.001 21122657

[8] Jiang C, Liu L, Li X, Ma L, Du L, Zhao Y, et al. Separation and purification of hypocholesterolaemic peptides from whey protein and their stability under simulated gastrointestinal digestion. Int J Dairy Technol 2018; 71(2): 460–8. doi: 10.1111/1471-0307.12453

[9] Jiang X, Pan D, Zhang T, Liu C, Zhang J, Su M, et al. Novel milk casein-derived peptides decrease cholesterol micellar solubility and cholesterol intestinal absorption in Caco-2 cells. J Dairy Sci 2020; 103(5): 3924–36. doi: 10.3168/jds.2019-17586 32113776

[10] Zhang H, Bartley GE, Zhang H, Jing W, Fagerquist CK, Zhong F, et al. Peptides identified in soybean protein increase plasma cholesterol in mice on hypercholesterolemic diets. J Agric Food Chem 2013; 61(35): 8389–95. doi: 10.1021/jf4022288 23937379

[11] Brandelli A, Daroit DJ, Corrêa APF. Whey as a source of peptides with remarkable biological activities. Food Res Int 2015; 73: 149–61. doi: 10.1016/j.foodres.2015.01.016

[12] Mann B, Athira S, Sharma R, Kumar R, Sarkar P. Bioactive Peptides from Whey Proteins. Whey Proteins 2019. p. 519–47. doi: 10.1016/B978-0-12-812124-5.00015-1

[13] Zhao C, Ashaolu TJ. Bioactivity and safety of whey peptides. LWT 2020; 134, 109935. doi: 10.1016/j.lwt.2020.109935

[14] Baba WN, Mudgil P, Baby B, Vijayan R, Gan CY, Maqsood S. New insights into the cholesterol esterase- and lipase-inhibiting potential of bioactive peptides from camel whey hydrolysates: identification, characterization, and molecular interaction. J Dairy Sci 2021; 104(7): 7393–405. doi: 10.3168/jds.2020-19868 33934858

[15] Morikawa K, Kondo I, Kanamaru Y, Nagaoka S. A novel regulatory pathway for cholesterol degradation via lactostatin. Biochem Biophys Res Commun 2007; 352(3): 697–702. doi: 10.1016/j.bbrc.2006.11.090 17141196

[16] Ding L, Wang L, Zhang Y, Liu J. Transport of antihypertensive peptide RVPSL, ovotransferrin 328–332, in human intestinal Caco-2 cell monolayers. J Agric Food Chem 2015; 63(37): 8143–50. doi: 10.1021/acs.jafc.5b01824 26335384

[17] Liu M, Zhang T, Liang X, Yuan Q, Zeng X, Wu Z, et al. Production and transepithelial transportation of casein-derived peptides and identification a novel antioxidant peptide LHSMK. LWT 2021; 151, 112194. doi: 10.1016/j.lwt.2021.112194

[18] Rao PS, Bajaj R, Mann B. Impact of sequential enzymatic hydrolysis on antioxidant activity and peptide profile of casein hydrolysate. J Food Sci Technol 2020; 57(12): 4562–75. doi: 10.1007/s13197-020-04495-2 33087969 PMC7550546

[19] Zhang T, Su M, Jiang X, Xue Y, Zhang J, Zeng X, et al. Transepithelial transport route and liposome encapsulation of milk-derived ACE-inhibitory peptide Arg-Leu-Ser-Phe-Asn-Pro. J Agric Food Chem 2019; 67(19): 5544–51. doi: 10.1021/acs.jafc.9b00397 31007021

[20] Ding L, Wang L, Yu Z, Zhang T, Liu J. Digestion and absorption of an egg white ACE-inhibitory peptide in human intestinal Caco-2 cell monolayers. Int J Food Sci Nutr 2016; 67(2): 111–6. doi: 10.3109/09637486.2016.1144722 26883099

[21] Segura-Campos M, Chel-Guerrero L, Betancur-Ancona D, Hernandez-Escalante VM. Bioavailability of bioactive peptides. Food Rev Int 2011; 27(3): 213–26. doi: 10.1080/87559129.2011.563395

[22] Ruiz JÁG, Ramos M, Recio I. Angiotensin converting enzyme-inhibitory activity of peptides isolated from Manchego cheese. Stability under simulated gastrointestinal digestion. Int Dairy J 2004; 14(12): 1075–80. doi: 10.1016/j.idairyj.2004.04.007

[23] Guo Y, Pan D, Tanokura M. Optimisation of hydrolysis conditions for the production of the angiotensin-I converting enzyme (ACE) inhibitory peptides from whey protein using response surface methodology. Food Chem 2009; 114(1): 328–33. doi: 10.1016/j.foodchem.2008.09.041

[24] Pan D, Guo Y. Optimization of sour milk fermentation for the production of ACE-inhibitory peptides and purification of a novel peptide from whey protein hydrolysate. Int Dairy J 2010; 20(7): 472–9. doi: 10.1016/j.idairyj.2010.01.007

[25] Wang X, Yu H, Xing R, Liu S, Chen X, Li P. Preparation and identification of antioxidative peptides from pacific herring (Clupea pallasii) protein. Molecules 2019; 24(10), 1946. doi: 10.3390/molecules24101946 31117172 PMC6572113

[26] Catanzaro D, Rancan S, Orso G, Dall’Acqua S, Brun P, Giron MC, et al. Boswellia serrata preserves intestinal epithelial barrier from oxidative and inflammatory damage. PLoS One 2015; 10(5): e0125375. doi: 10.1371/journal.pone.0125375 25955295 PMC4425476

[27] Ketnawa S, Ogawa Y. In vitro protein digestibility and biochemical characteristics of soaked, boiled and fermented soybeans. Sci Rep 2021; 11(1): 14257. doi: 10.1038/s41598-021-93451-x 34244542 PMC8270925

[28] Golneshin A, Gor MC, Williamson N, Vezina B, Van TTH, May BK, et al. Discovery and characterisation of circular bacteriocin plantacyclin B21AG from Lactiplantibacillus plantarum B21. Heliyon 2020; 6(8): e04715. doi: 10.1016/j.heliyon.2020.e04715 32904251 PMC7452424

[29] Guo Y, Jiang X, Xiong B, Zhang T, Zeng X, Wu Z, et al. Production and transepithelial transportation of angiotensin-I-converting enzyme (ACE)-inhibitory peptides from whey protein hydrolyzed by immobilized Lactobacillus helveticus proteinase. J Dairy Sci 2019; 102(2): 961–75. doi: 10.3168/jds.2018-14899 30594363

[30] Zhang HY, Yi JQ, Piao XS, Li PF, Zeng ZK, Wang D, et al. The metabolizable energy value, standardized ileal digestibility of amino acids in soybean meal, soy protein concentrate and fermented soybean meal, and the application of these products in early-weaned piglets. Asian-Australas J Anim Sci 2013; 26(5): 691–9. doi: 10.5713/ajas.2012.12429 25049840 PMC4093336

[31] Wang J, Luo D, Liang M, Zhang T, Yin X, Zhang Y, et al. Spectrum-effect relationships between high-performance liquid chromatography (HPLC) fingerprints and the antioxidant and anti-inflammatory activities of collagen peptides. Molecules 2018; 23(12), 3257. doi: 10.3390/molecules23123257 30544714 PMC6320860

[32] Karaś M. Influence of physiological and chemical factors on the absorption of bioactive peptides. Int J Food Sci Technol 2018; 54(5): 1486–96. doi: 10.1111/ijfs.14054

[33] Shimizu M, Tsunogai M, Arai S. Transepithelial transport of oligopeptides in the human intestinal cell, Caco-2. Peptides 1997; 18: 681–687. doi: 10.1016/S0196-9781(97)00002-8 9213361

[34] Hymel HC, Rahnama A, Sanchez OM, Liu D, Gauthier TJ, Melvin AT. How cargo identity alters the uptake of cell-penetrating peptide (CPP)/cargo complexes: a study on the effect of net cargo charge and length. Cells 2022; 11(7), 1195. doi: 10.3390/cells11071195 35406759 PMC8997848

[35] Fernandez-Musoles R, Salom JB, Martinez-Maqueda D, Lopez-Diez JJ, Recio I, Manzanares P. Antihypertensive effects of lactoferrin hydrolyzates: inhibition of angiotensin- and endothelin-converting enzymes. Food Chem 2013; 139(1–4): 994–1000. doi: 10.1016/j.foodchem.2012.12.049 23561201

[36] Zhuang MZ, Zhao MM, Lin LZ, Dong Y, Chen HP, Feng MY, et al. Macroporous resin purification of peptides with umami taste from soy sauce. Food Chem 2016; 190: 338–44. doi: 10.1016/j.foodchem.2015.05.105 26212979

[37] Pak VV, Koo MS, Kasymova TD, Kwon DY. Isolation and identification of peptides from soy 11S-globulin with hypocholesterolemic activity. Chem Nat Compd 2005; 41:710–714. doi: 10.1007/s10600-006-0017-6

[38] Yoshikawa M, Fujita H, Matoba N, Takenaka Y, Yamamoto T, Yamauchi R, et al. Bioactive peptides derived from food proteins preventing lifestyle-related diseases. Biofactors 2000; 12: 143–146. doi: 10.1002/biof.5520120122 11216476

[39] Wang J, Shimada M, Kato Y, Kusada M, Nagaoka S. Cholesterol-lowering effect of rice bran protein containing bile acid-binding proteins. Biosci Biotech Bioch 2015; 79(3): 456–61. doi: 10.1080/09168451.2014.978260 25374002

[40] Lapphanichayakool P, Sutheerawattananonda M, Limpeanchob N. Hypocholesterolemic effect of sericin-derived oligopeptides in high-cholesterol fed rats. J Nat Med 2017; 71(1): 208–15. doi: 10.1007/s11418-016-1050-9 27771849

[41] Yuan X, Bao X, Liu X, Li X. Flaxseed-derived peptides ameliorate hepatic cholesterol metabolism in Sprague-Dawley rats fed a high-cholesterol and high-fat diet. J Sci Food Agric 2022; 102(12): 5348–57. doi: 10.1002/jsfa.11888 35318649

[42] Ohsawa K, Satsu H, Ohki K, Enjoh M, Takano T, Shimizu M. Producibility and digestibility of antihypertensive *β*-casein tripeptides, Val-Pro-Pro and Ile-Pro-Pro, in the gastrointestinal tract: analyses using an in vitro model of mammalian gastrointestinal digestion. J Agric Food Chem 2008; 56: 854–858. doi: 10.1021/jf072671n 18193838

[43] Chen M, Li B. The effect of molecular weights on the survivability of casein-derived antioxidant peptides after the simulated gastrointestinal digestion. Innov Food Sci Emerg Technol 2012; 16: 341–8. doi: 10.1016/j.ifset.2012.07.009

