# Supplementary material for: Transportation of whey protein-derived peptides using Caco-2 cell model and identification of novel cholesterol-lowering peptides
Source: Food Nutr Res. 2023 May 25;67:10.29219/fnr.v67.9079. doi: 10.29219/fnr.v67.9079 (PMC10243119; doi:10.29219/fnr.v67.9079)
Supplement: Supplementary file 1 [file FNR-67-9079-s001.docx]

**Supplementary material**

**Fig. S1.** Establishment of a monolayer model of intestinal Caco-2 cells for index determination.

**Fig. S2.** Tandem mass spectra of six novel peptides from fraction 1 of alkaline protease hydrolysate.

**Fig. S3.** Tandem mass spectra of six novel peptides from fraction 2 of alkaline protease hydrolysate.

**Fig. S4.** Tandem mass spectra of five novel peptides from fraction 3 of trypsin protease hydrolysate.

**Fig. S5.** Tandem mass spectra of five novel peptides from fraction 4 of trypsin hydrolysate.

Fig. S1. Establishment of a monolayer model of intestinal Caco-2 cells for index determination .Verifying the construction of the Caco-2 cell model, A is the observation of the Caco-2 cell monolayer under a transmission electron microscope (20000×); B is the Caco-2 cell model monolayer compactness measurement diagram, which measures transepithelial resistance (TEER) values; C is the Caco-2 cell model monolayer polarity measurement diagram, which determines the AP to BL side alkaline phosphatase ratio; D is the monolayer integrity measurement of the Caco-2 cell model, which determines the permeability of sodium fluorescein.

Fig. S2: Tandem mass spectra of six novel peptides from fraction 1 of alkaline protease hydrolysate; A to F represent LVG, ALK, FDK, F, AH and GLF in that order.

Fig. S3: Tandem mass spectra of six novel peptides from fraction 2 of alkaline protease hydrolysate; A to F represent ALK, FDK, AEK, AVFK, EV, and AH in the order.

Fig. S4: Tandem mass spectra of five novel peptides from fraction 3 of trypsin protease hydrolysate; A to E represent ALK, LK, F, ALPM, and GLF in the order.

Fig. S5: Tandem mass spectra of five novel peptides from fraction 4 of trypsin hydrolysate; A to D represent KDLK, KLD, LK and VEE in the order.
